# Bis(acetylacetonato)copper(II) – structural and electronic data of the neutral, oxidized and reduced forms

**DOI:** 10.1016/j.dib.2019.104511

**Published:** 2019-09-13

**Authors:** Jeanet Conradie

**Affiliations:** Department of Chemistry, PO Box 339, University of the Free State, Bloemfontein, 9300, South Africa

**Keywords:** Cupric acetylacetonate, Cu(acac)_2_, DFT, Reduction, Molecular orbital

## Abstract

Bis(acetylacetonato)copper(II) can be synthesized economically and with ease by the reaction between acetylacetone and a copper salt (Cu(OAc)_2_ or CuCl_2_·2H_2_O). When used as catalyst, bis(acetylacetonato)copper(II) is sometimes being oxidized to Cu(III) or reduced to Cu(I), although only the structure of the neutral form is known experimentally. The content of this paper provides computational chemistry calculated data of the geometry, electronic structure, spin state and frontier orbitals for the neutral, as well as the oxidized and reduced forms of the bis(acetylacetonato)copper(II) molecule. This data shows that both the highest occupied molecular orbital (HOMO) and the lowest unoccupied molecular orbital (LUMO) of the neutral molecule are copper based. The neutral molecule is a spin = ½ system. The data shows that the spin state of both the oxidized and reduced molecules is zero.

**Table:** Specifications Table

Subject area	Chemistry
More specific subject area	Computational chemistry
Type of data	Table, text file, graph, figure
How data was acquired	Electronic structure calculations, using the Amsterdam Density Functional (ADF) 2016 programme.
Data format	Raw and Analyzed Data
Experimental factors	Data were collected from DFT output files and from the Cambridge Structural Database (CSD).
Experimental features	DFT data was obtained with the Amsterdam Density Functional (ADF) 2016 programme on the High Performance Computing facility of the University of the Free State
Data source location	Department of Chemistry, University of the Free State, Nelson Mandela Street, Bloemfontein, South Africa
Data accessibility	Data is included with article
Related research article	E. Chiyindiko, J. Conradie, Redox behaviour of bis(β-diketonato)copper(II) complexes, Journal of Electroanalytical Chemistry 837 (2019) 76–85. https://doi.org/10.1016/j.jelechem.2019.02.011.

**Table:** 

**Value of the Data** • This data can be used to visualize the density functional theory calculated optimized structures, for the neutral, oxidized and reduced forms of [Cu(acac)_2_] • This data can be used to determine the density functional theory calculated lowest energy spin states of the neutral, oxidized and reduced forms of [Cu(acac)_2_] • This data visualizes the density functional theory calculated Cu-d-based frontier orbitals, for the neutral, oxidized and reduced forms of [Cu(acac)_2_] • This data provides density functional theory calculated molecular orbital energy level diagrams, for the neutral, oxidized and reduced forms of [Cu(acac)_2_] • This data can be used to understand the change in electron occupation and frontier molecular orbital energies, during reduction and oxidation of [Cu(acac)_2_]

## 1 Data

**Fig. 1:**
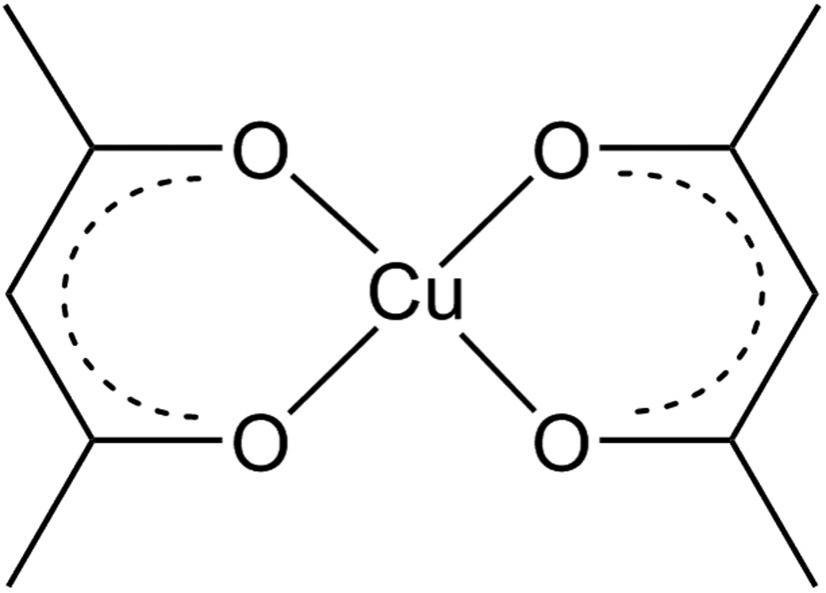
Structure of bis(acetylacetonato)copper(II), [Cu(acac)_2_].

**Fig. 2:**
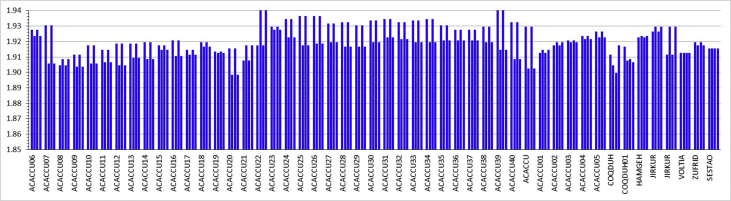
Experimental data: The four Cu–O bond lengths (in Å, given on the y-axis) found in each of 49 different crystals of bis(acetylacetonato)copper(II), [Cu(acac)_2_], with the respective CSD code [4] indicated on the x-axis. Data provided in the Supplementary Information.

**Fig. 3:**
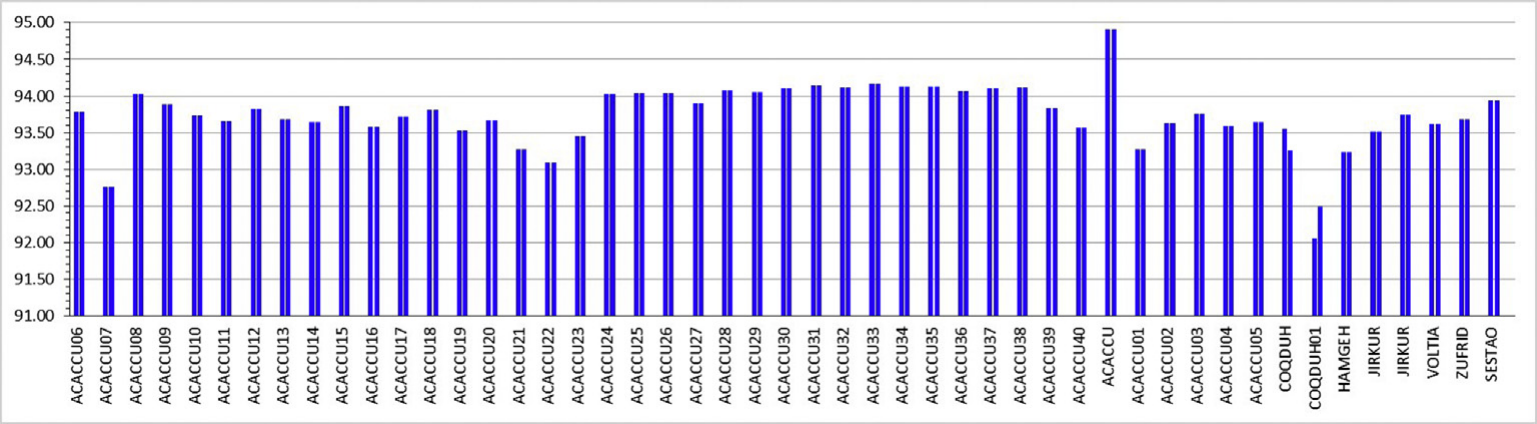
Experimental data: The two O–Cu–O bond angles (in degrees, given on the y-axis) found in each of 49 different crystals of bis(acetylacetonato)copper(II), [Cu(acac)_2_], with the respective CSD code [4] indicated on the x-axis. Data provided in the Supplementary Information.

Fig. 1 shows the structure of the molecule bis(acetylacetonato)copper(II), [Cu(acac)_2_]. The neutral d^9^ molecule has a spin state of ½, therefore contains one unpaired electron, as is expected for Cu(II) complexes [1], [2], [3]. Fig. 2 shows the four Cu–O bond lengths within molecule [Cu^II^(acac)_2_], for each of 49 different crystals obtained from the CSD [4]. The experimentally obtained Cu–O bond lengths vary between 1.898 and 1.942 Å, with an average of 1.919(9) Å. The experimentally obtained O–Cu–O bond angles vary between 92.0 and 94.9°, with an average of 93.7(4)°, see Fig. 3. Table 1 compares the average of the experimental data with DFT calculated geometrical data involving Cu.

**Table 1:** Averages of the experimental (Exp) and DFT calculated (Calc) geometrical parameters for ([Cu^II^(acac)_2_]).

Functional	O–Cu–O bond angle (deg)	[Calc—Exp]	Cu–O bond length (Å)	[Calc—Exp]
**Exp. Parameters:**				
Experimental range	92.0–94.9		1.898–1.942	
Experimental average	93.7(4)	–	1.919(9)	–
**Calc. Parameters:**				
OLYP	92.4	−1.4	1.977	0.057
BP86	93.6	−0.1	1.948	0.029
B3LYP	93.0	−0.7	1.940	0.021
O3LYP	94.2	0.5	1.862	−0.057
M06-L	91.1	−2.6	1.943	0.024
BLYP	92.9	−0.8	1.971	0.052
B3LYP*	93.2	−0.5	1.941	0.022

**Fig. 4:**
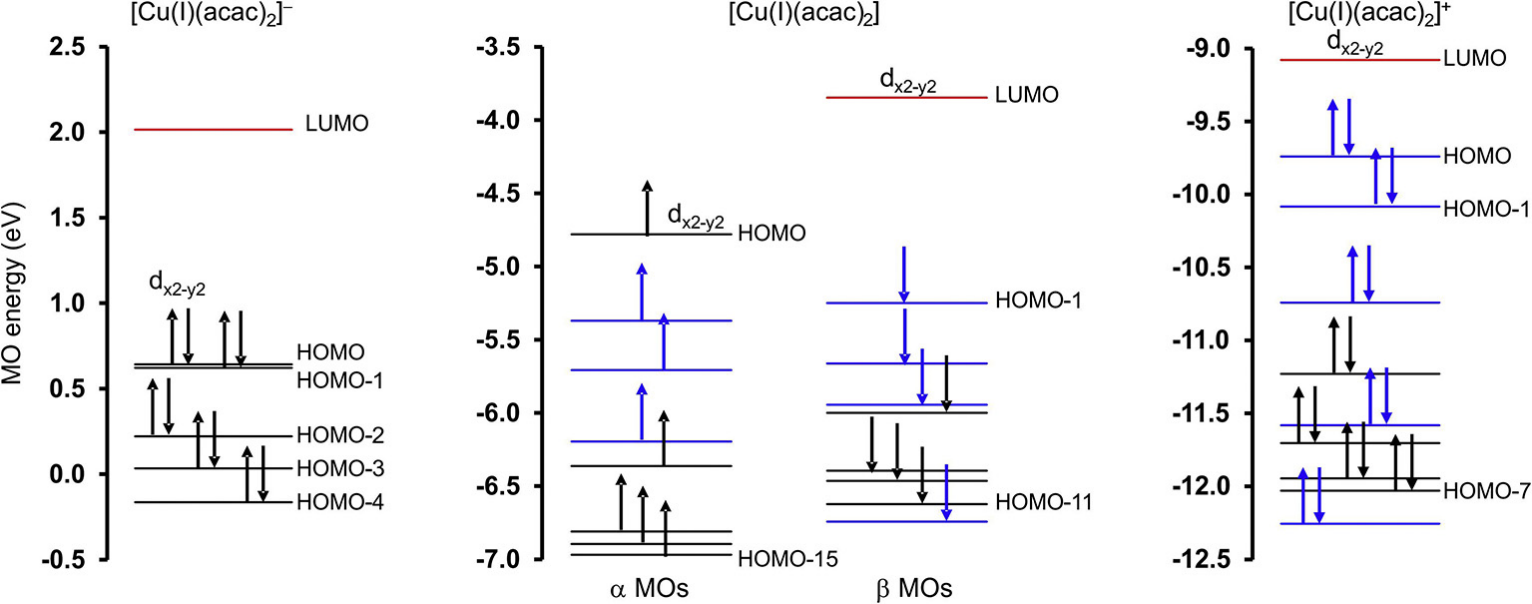
The OLYP/TZP Kohn-Sham MO energy level (in eV, on the y-axis) diagrams, for all three forms of [Cu(acac)_2_], namely the reduced (anion, left), neutral (middle) and oxidized (cation, right) forms. The energy levels of filled MOs are shown in black (for Cu-d antibonding MOs) or blue (for ligand based MOs), and the energy levels of empty MOs in red. The arrows indicate the α-electrons (up spin) and β electrons (down spin).

**Fig. 5:**
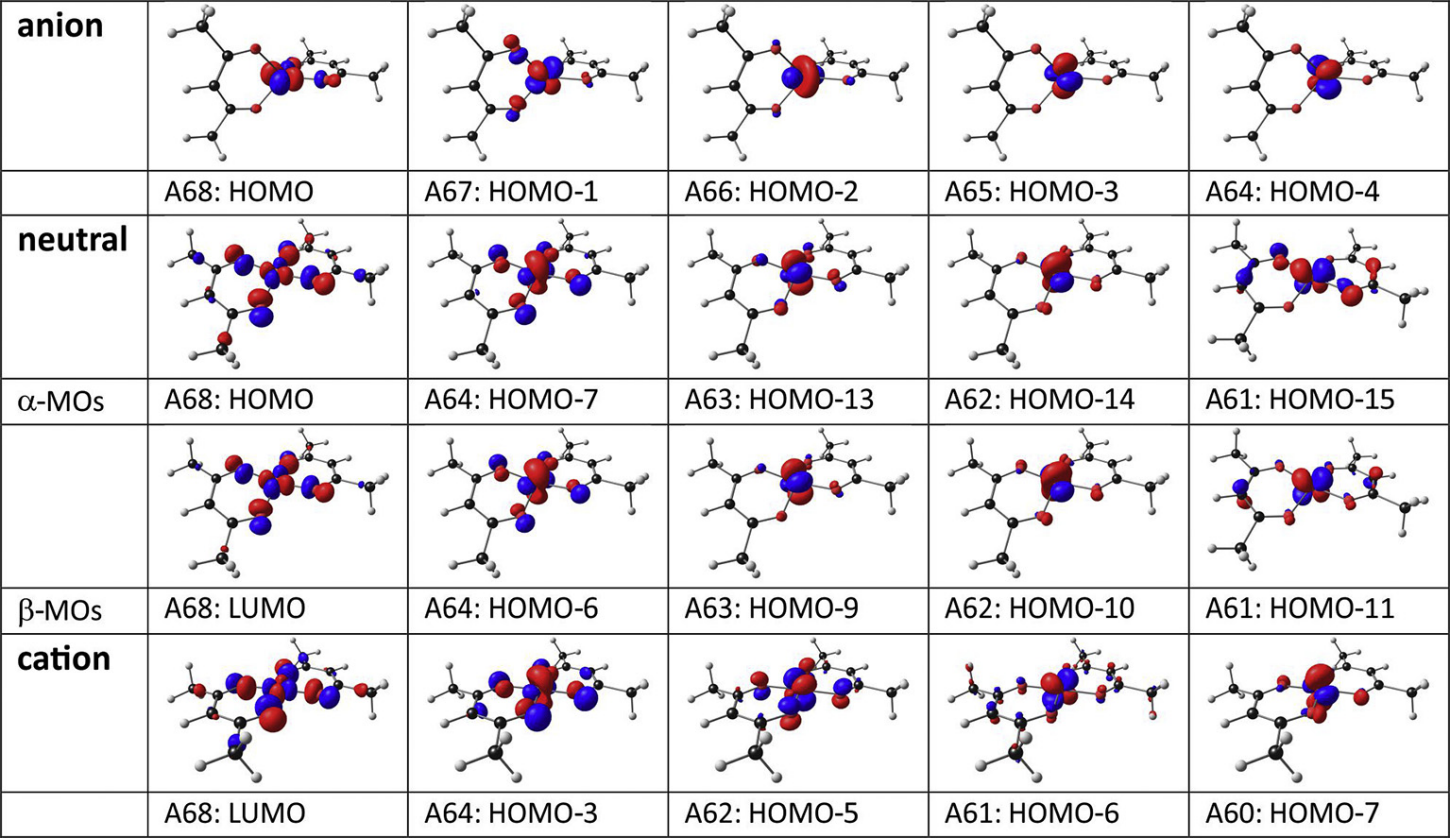
The OLYP/TZP metal d-based anti-bonding MOs for all three forms of [Cu(acac)_2_] complex, namely the reduced (anion, top), neutral (middle) and oxidized (cation, bottom) forms. Contour = 0.06 e/Å^3^.

Fig. 4 (middle) shows a Kohn-Sham molecular orbital (MO) energy level diagram for molecule [Cu(acac)_2_], which has a d-occupation of $d_{xy}^{2}d_{xz}^{2}d_{yz}^{2}d_{z^{2}}^{2}d_{x^{2}-y^{2}}^{1}$. The data in Table 2 shows that the spin state of both the oxidized ([Cu^III^(acac)_2_]^+^) and reduced ([Cu^I^(acac)_2_]^-^) molecules is zero (closed shell singlets), therefore no unpaired electrons exist in either of these forms. Also shown in Fig. 4, are the Kohn-Sham MO energy level diagrams (in eV) of the reduced molecule ([Cu^I^(acac)_2_]^-^, left diagram) and oxidized molecule ([Cu^III^(acac)_2_]^+^, right diagram), which illustrate the change both in electron occupation, as well as in frontier molecular orbital energies, during reduction and oxidation of [Cu(acac)_2_] respectively. The Cu-d-based antibonding orbitals of all three forms of [Cu^II^(acac)_2_], namely the reduced (anion), neutral and oxidized (cation) forms, are shown in Fig. 5.

**Table 2:** The OLYP/TZP calculated relative energies (ΔE) for different spin states (S), for both the oxidized ([Cu^III^(acac)_2_]^+^) and reduced ([Cu^I^(acac)_2_]^-^) molecules.

	S	ΔE (eV)
[Cu^III^(acac)_2_]^+^	0	0.00
	1	0.45
[Cu^I^(acac)_2_]^-^	0	0.00
	1	1.45

## 2 Experimental design, materials, and methods

Density functional theory (DFT) calculations were performed in the gas phase on the neutral, oxidized and reduced forms of the molecule, using the Amsterdam Density Functional (ADF) 2016 programme [5]. Seven different functionals in combination with the TZP (Triple ζ polarized) basis set were used, namely: OLYP (Handy-Cohen and Lee-Yang-Parr) [6], [7], [8], [9], B3LYP [7], [10], B3LYP* [11], O3LYP [12], BLYP [7], [8], [9], [13], BP86 [13], [14] and M06-L [15], [16]]. Input coordinates were constructed theoretically, using ChemCraft [17]. ChemCraft was also used to visualize the ADF output files. Experimental crystal structural data was obtained using ConQuest Version 1.21, to search for existing [Cu^II^(acac)_2_] crystal structures in the Cambridge Structural Database [4]. The optimized coordinates, as well as an example input file, are provided in the supplementary information.
