# Supplementary material for: Bis(acetylacetonato)copper(II) – structural and electronic data of the neutral, oxidized and reduced forms
Source: Data Brief. 2019 Sep 13;26:104511. doi: 10.1016/j.dib.2019.104511 (PMC6811892; doi:10.1016/j.dib.2019.104511)
Supplement: Multimedia component 1 [file mmc1.docx]

**Data Article**

**Title**: Bis(acetylacetonato)copper(II) – structural and electronic data of the neutral, oxidized and reduced forms.

**Authors**: Jeanet Conradie

**Affiliations**: Department of Chemistry, PO Box 339, University of the Free State, Bloemfontein, 9300, South Africa

**Contact email**: conradj@ufs.ac.za

Supporting information

Table of Contents

[**Data Article** 1](#_Toc453550)

[Example input file 2](#_Toc453551)

[Optimized Cartesian coordinates (Å) 4](#_Toc453552)

[1. [Cu^II^(acac)_2_] S = ½ OLYP/TZP 4](#_Toc453553)

[2. [Cu^III^(acac)_2_]^+^ S = 0 OLYP/TZP 4](#_Toc453554)

[3. [Cu^III^(acac)_2_]^+^ S = 1 OLYP/TZP 5](#_Toc453555)

[4. [Cu^I^(acac)_2_]^-^ S = 0 OLYP/TZP 6](#_Toc453556)

[5. [Cu^I^(acac)_2_]^-^ S = 1 OLYP/TZP 6](#_Toc453557)

[6. [Cu^II^(acac)_2_] S = ½ BP86/TZP 7](#_Toc453558)

[7. [Cu^II^(acac)_2_] S = ½ B3LYP/TZP 7](#_Toc453559)

[8. [Cu^II^(acac)_2_] S = ½ O3LYP/TZP 8](#_Toc453560)

[9. [Cu^II^(acac)_2_] S = ½ M06-L/TZP 9](#_Toc453561)

[10. [Cu^II^(acac)_2_] S = ½ BLYP/TZP 9](#_Toc453562)

[11. [Cu^II^(acac)_2_] S = ½ B3LYP*/TZP 10](#_Toc453563)

# Example input file

Title Cu(II)acac2

COMMENT

Optimization of Cu(II)acac2 spin = 1/2

END

UNITS

length angstrom

END

atoms cartesian

Cu -0.107726000 0.362008000 0.385822000

O -1.652802000 -0.286514000 -1.280355000

O 0.195506000 2.042334000 -0.570691000

C -1.299752000 1.786480000 -2.472510000

H -1.621750000 2.316298000 -3.368525000

C 0.154514000 3.820813000 -2.135386000

H 1.222243000 3.737870000 -2.430798000

H 0.120721000 4.554950000 -1.318352000

H -0.403927000 4.222592000 -2.990733000

C -1.862487000 0.495189000 -2.271258000

C -2.907498000 0.018662000 -3.289908000

H -3.911479000 -0.024519000 -2.816595000

H -2.676625000 -1.006008000 -3.609162000

H -2.977236000 0.657105000 -4.182521000

C -0.371243000 2.469614000 -1.659099000

O 2.736228000 -1.359391000 0.535899000

O -0.115196000 -0.761431000 1.959921000

C 1.725511000 -2.276082000 2.531044000

H 1.954855000 -3.004243000 3.307518000

C -0.352777000 -2.060994000 3.914513000

H -0.669392000 -1.175860000 4.481479000

H -1.277610000 -2.565278000 3.578102000

H 0.184084000 -2.738103000 4.591736000

C 2.725287000 -2.148622000 1.511928000

C 3.972615000 -3.058633000 1.676872000

H 4.142564000 -3.612931000 0.744539000

H 4.867176000 -2.427397000 1.832513000

H 3.908550000 -3.778244000 2.505785000

C 0.479220000 -1.629043000 2.696927000

end

SYMMETRY nosym

CHARGE -2 1

unrestricted

INTEGRATION 6.0

GEOMETRY

Iterations 200

Convergence e=0.001 grad=0.001 rad=0.01

END

SCF

Iterations 1000

Convergens 1e-6

END

BASIS

Type TZP

Core None

END

XC

gga OLYP

end

endinput

# Optimized Cartesian coordinates (Å)

# [Cu^II^(acac)_2_] S = ½ OLYP/TZP

Cu -0.002411000 -0.005079000 -0.005709000

O -1.709460000 0.987372000 -0.095110000

O 0.783789000 1.051427000 -1.479070000

C -1.100510000 2.472521000 -1.868456000

H -1.460793000 3.291945000 -2.479457000

C 1.054577000 2.669896000 -3.171738000

H 1.349674000 1.917666000 -3.911634000

H 1.975746000 3.052616000 -2.718625000

H 0.532259000 3.485923000 -3.673975000

C -1.975450000 1.943156000 -0.901910000

C -3.372525000 2.518062000 -0.764497000

H -3.523048000 2.859703000 0.265377000

H -4.106297000 1.725819000 -0.949895000

H -3.554345000 3.346234000 -1.451789000

C 0.208588000 2.010595000 -2.099079000

O 1.642971000 -1.101312000 -0.013225000

O -0.727423000 -0.957441000 1.566685000

C 1.107459000 -2.458610000 1.882619000

H 1.473767000 -3.266375000 2.505435000

C -0.923359000 -2.444388000 3.384893000

H -1.094945000 -1.631680000 4.099277000

H -1.907149000 -2.788085000 3.046673000

H -0.406302000 -3.263594000 3.887591000

C 1.918626000 -2.038329000 0.812311000

C 3.251745000 -2.722265000 0.575702000

H 3.278503000 -3.117735000 -0.445438000

H 4.055428000 -1.981545000 0.654412000

H 3.436873000 -3.532819000 1.282800000

C -0.147159000 -1.905641000 2.198413000

# [Cu^III^(acac)_2_]^+^ S = 0 OLYP/TZP

Cu -0.002941000 -0.000993000 0.000303000

O -1.610278000 0.989833000 -0.014031000

O 0.733756000 0.927076000 -1.469627000

C -1.082894000 2.432075000 -1.845225000

H -1.440480000 3.250403000 -2.456377000

C 1.029725000 2.432088000 -3.244052000

H 1.189195000 1.636509000 -3.980250000

H 2.011131000 2.719006000 -2.852367000

H 0.555414000 3.288167000 -3.723813000

C -1.901919000 1.954781000 -0.815813000

C -3.263391000 2.561878000 -0.562788000

H -3.342145000 2.849164000 0.490505000

H -4.032175000 1.805073000 -0.754947000

H -3.434543000 3.429449000 -1.200392000

C 0.179806000 1.893466000 -2.115747000

O 1.618812000 -0.967480000 0.032756000

O -0.750175000 -0.954781000 1.448491000

C 1.088477000 -2.428315000 1.846742000

H 1.450326000 -3.243315000 2.459800000

C -1.058975000 -2.498914000 3.187769000

H -1.267846000 -1.718355000 3.927516000

H -2.017567000 -2.812855000 2.761636000

H -0.572562000 -3.345393000 3.672419000

C 1.919725000 -1.924164000 0.840440000

C 3.303905000 -2.491080000 0.618352000

H 3.419548000 -2.765992000 -0.434807000

H 4.045135000 -1.714360000 0.838056000

H 3.482929000 -3.359148000 1.253128000

C -0.192414000 -1.921008000 2.091939000

# [Cu^III^(acac)_2_]^+^ S = 1 OLYP/TZP

Cu 0.004798000 0.005372000 0.004415000

O -1.682691000 1.003212000 -0.094697000

O 0.764327000 1.056450000 -1.469165000

C -1.110850000 2.465114000 -1.885875000

H -1.477582000 3.274358000 -2.507665000

C 1.058940000 2.652490000 -3.189293000

H 1.364664000 1.890025000 -3.914814000

H 1.975879000 3.044532000 -2.734813000

H 0.534009000 3.457001000 -3.704870000

C -1.993933000 1.943634000 -0.896890000

C -3.385836000 2.503024000 -0.760291000

H -3.543060000 2.830889000 0.273156000

H -4.110928000 1.703838000 -0.953506000

H -3.571495000 3.333646000 -1.441901000

C 0.217932000 2.006872000 -2.119449000

O 1.638731000 -1.083403000 0.019626000

O -0.706359000 -0.959194000 1.558689000

C 1.103156000 -2.473782000 1.878243000

H 1.462574000 -3.292657000 2.491636000

C -0.945443000 -2.450839000 3.377430000

H -1.089275000 -1.645262000 4.106967000

H -1.941950000 -2.754936000 3.038044000

H -0.449241000 -3.293081000 3.860264000

C 1.934937000 -2.042664000 0.804698000

C 3.250267000 -2.730957000 0.550699000

H 3.277224000 -3.081403000 -0.486943000

H 4.062518000 -2.003094000 0.661706000

H 3.419727000 -3.570223000 1.225925000

C -0.165699000 -1.914874000 2.205604000

# [Cu^I^(acac)_2_]^-^ S = 0 OLYP/TZP

Cu 0.237825000 -0.006807000 0.170665000

O -1.170072000 -0.432683000 -1.442881000

O 0.342683000 1.986966000 -0.514825000

C -1.310756000 1.823747000 -2.255081000

H -1.831640000 2.430869000 -2.990635000

C -0.174785000 3.997635000 -1.662450000

H 0.885668000 4.199187000 -1.856907000

H -0.440664000 4.537952000 -0.745080000

H -0.771312000 4.391821000 -2.491563000

C -1.652142000 0.446037000 -2.211240000

C -2.719606000 -0.046859000 -3.200110000

H -3.529452000 -0.530612000 -2.640537000

H -2.279001000 -0.812061000 -3.851811000

H -3.141322000 0.749758000 -3.822071000

C -0.367943000 2.492491000 -1.434016000

O 1.979944000 -1.267122000 0.348612000

O -0.314189000 -0.534083000 2.146552000

C 1.509460000 -2.041591000 2.575543000

H 1.900218000 -2.705068000 3.342328000

C -0.318798000 -1.609106000 4.260809000

H -0.440261000 -0.655369000 4.788967000

H -1.323576000 -2.027525000 4.120499000

H 0.265009000 -2.291672000 4.886804000

C 2.249993000 -1.965038000 1.367846000

C 3.526644000 -2.813581000 1.271774000

H 3.488717000 -3.417866000 0.357528000

H 4.394886000 -2.148138000 1.181126000

H 3.676359000 -3.473431000 2.132535000

C 0.311585000 -1.349279000 2.886194000

# [Cu^I^(acac)_2_]^-^ S = 1 OLYP/TZP

Cu 0.163543000 -0.075214000 0.152046000

O -1.683789000 0.065473000 -0.567639000

O 0.893058000 1.322948000 -1.054267000

C -1.194760000 1.806681000 -2.166991000

H -1.644099000 2.434855000 -2.932396000

C 0.959802000 3.020725000 -2.706316000

H 1.760360000 2.536332000 -3.286634000

H 1.455856000 3.724231000 -2.021045000

H 0.324751000 3.588945000 -3.393673000

C -2.036781000 0.885970000 -1.515843000

C -3.500034000 0.796667000 -1.904326000

H -4.140034000 1.015149000 -1.035748000

H -3.746693000 -0.227683000 -2.222050000

H -3.758997000 1.487114000 -2.713555000

C 0.178984000 1.985256000 -1.918929000

O 2.032617000 -0.318121000 0.783540000

O -0.593951000 -1.380326000 1.447845000

C 1.509456000 -1.939673000 2.493910000

H 1.955858000 -2.557315000 3.269508000

C -0.695946000 -2.936178000 3.232465000

H -1.444061000 -2.352398000 3.790547000

H -1.257215000 -3.666459000 2.630286000

H -0.069869000 -3.477789000 3.948573000

C 2.379590000 -1.129549000 1.740807000

C 3.868760000 -1.155900000 2.025962000

H 4.423438000 -1.464022000 1.126261000

H 4.225566000 -0.146681000 2.281027000

H 4.124166000 -1.836502000 2.844538000

C 0.114120000 -2.024716000 2.330716000

# [Cu^II^(acac)_2_] S = ½ BP86/TZP

Cu 0.002043000 0.003061000 0.002885000

O -1.689855000 0.966232000 -0.073039000

O 0.798335000 1.038750000 -1.442609000

C -1.098085000 2.435946000 -1.867323000

H -1.466000000 3.247463000 -2.492538000

C 1.060825000 2.638137000 -3.156279000

H 1.376944000 1.876025000 -3.882032000

H 1.973503000 3.045371000 -2.699254000

H 0.525463000 3.439053000 -3.677777000

C -1.967055000 1.914339000 -0.893374000

C -3.362240000 2.482545000 -0.756092000

H -3.507577000 2.841379000 0.272166000

H -4.096831000 1.682567000 -0.924392000

H -3.548737000 3.301650000 -1.459336000

C 0.215767000 1.986019000 -2.084560000

O 1.630155000 -1.066957000 -0.023160000

O -0.730285000 -0.924665000 1.551574000

C 1.091759000 -2.446241000 1.857457000

H 1.453794000 -3.266719000 2.474379000

C -0.935166000 -2.425928000 3.358807000

H -1.082175000 -1.620713000 4.092320000

H -1.932452000 -2.740871000 3.022151000

H -0.428024000 -3.268363000 3.841541000

C 1.904029000 -2.020134000 0.792376000

C 3.228199000 -2.707116000 0.541973000

H 3.245239000 -3.097301000 -0.485055000

H 4.038807000 -1.969089000 0.619365000

H 3.412207000 -3.525226000 1.246930000

C -0.156867000 -1.886825000 2.179200000

# [Cu^II^(acac)_2_] S = ½ B3LYP/TZP

Cu -0.000237000 -0.001135000 -0.001473000

O -1.628992000 1.052557000 0.001223000

O 0.734748000 0.951104000 -1.522717000

C -1.092920000 2.436386000 -1.857560000

H -1.456192000 3.244769000 -2.474624000

C 0.959147000 2.460924000 -3.309914000

H 1.162512000 1.660892000 -4.023773000

H 1.923246000 2.816738000 -2.942923000

H 0.440007000 3.273179000 -3.814285000

C -1.911229000 1.993619000 -0.810726000

C -3.250205000 2.649846000 -0.582763000

H -3.288045000 3.039652000 0.435678000

H -4.035318000 1.896467000 -0.667039000

H -3.443609000 3.455191000 -1.288019000

C 0.166802000 1.901332000 -2.154603000

O 1.667234000 -0.990966000 0.058328000

O -0.772076000 -1.012139000 1.462543000

C 1.091447000 -2.437088000 1.856531000

H 1.453658000 -3.246109000 2.473456000

C -1.036080000 -2.587716000 3.186820000

H -1.304103000 -1.821850000 3.916656000

H -1.966760000 -2.960739000 2.756277000

H -0.516628000 -3.400758000 3.689479000

C 1.947721000 -1.932769000 0.870112000

C 3.328410000 -2.519759000 0.712258000

H 3.448138000 -2.883378000 -0.309519000

H 4.069208000 -1.732235000 0.861181000

H 3.517304000 -3.331134000 1.411955000

C -0.203351000 -1.960459000 2.096346000

# [Cu^II^(acac)_2_] S = ½ O3LYP/TZP

Cu -0.004900000 -0.007119000 -0.006550000

O -1.590554000 0.968710000 -0.013586000

O 0.735069000 0.923475000 -1.438877000

C -1.058351000 2.379563000 -1.800943000

H -1.416856000 3.194683000 -2.411584000

C 0.997507000 2.439061000 -3.153421000

H 1.239980000 1.651632000 -3.866104000

H 1.943530000 2.794623000 -2.746899000

H 0.487121000 3.251118000 -3.662155000

C -1.876078000 1.904842000 -0.799558000

C -3.199794000 2.510561000 -0.593758000

H -3.269233000 2.872815000 0.431132000

H -3.962316000 1.740467000 -0.704783000

H -3.395430000 3.323192000 -1.286532000

C 0.193021000 1.867652000 -2.063512000

O 1.586247000 -0.974242000 0.007754000

O -0.746279000 -0.942342000 1.422195000

C 1.068439000 -2.364104000 1.815454000

H 1.436982000 -3.163214000 2.441068000

C -0.992437000 -2.434593000 3.159345000

H -1.240836000 -1.641189000 3.863411000

H -1.935693000 -2.802005000 2.757182000

H -0.475126000 -3.236430000 3.677216000

C 1.882455000 -1.894225000 0.808721000

C 3.216137000 -2.482615000 0.617485000

H 3.296995000 -2.855812000 -0.402594000

H 3.966355000 -1.699752000 0.723573000

H 3.420110000 -3.283747000 1.321196000

C -0.192545000 -1.868579000 2.063284000

# [Cu^II^(acac)_2_] S = ½ M06-L/TZP

Cu 0.002309000 -0.001457000 -0.003158000

O -1.624337000 1.060167000 -0.032030000

O 0.710512000 0.978077000 -1.525261000

C -1.104919000 2.456515000 -1.871813000

H -1.469798000 3.265691000 -2.486225000

C 0.946056000 2.480164000 -3.293840000

H 1.162867000 1.686372000 -4.006381000

H 1.905912000 2.834494000 -2.922466000

H 0.437803000 3.291703000 -3.805246000

C -1.911748000 2.004205000 -0.834556000

C -3.240410000 2.643043000 -0.598864000

H -3.283927000 3.024009000 0.419736000

H -4.024652000 1.893064000 -0.684165000

H -3.442741000 3.452622000 -1.293325000

C 0.148137000 1.927197000 -2.159669000

O 1.659750000 -1.013524000 0.081104000

O -0.744425000 -1.026332000 1.469293000

C 1.098869000 -2.454461000 1.873111000

H 1.460709000 -3.261773000 2.491892000

C -1.013798000 -2.578441000 3.191101000

H -1.283257000 -1.811405000 3.914936000

H -1.946205000 -2.949420000 2.769704000

H -0.504286000 -3.389183000 3.702700000

C 1.942090000 -1.956439000 0.887301000

C 3.305808000 -2.540889000 0.718645000

H 3.421825000 -2.907559000 -0.299416000

H 4.053975000 -1.762245000 0.856084000

H 3.501214000 -3.350060000 1.415750000

C -0.183305000 -1.972473000 2.108159000

# [Cu^II^(acac)_2_] S = ½ BLYP/TZP

Cu -0.000495000 -0.000099000 0.000139000

O -1.666734000 1.053074000 -0.017486000

O 0.752923000 0.980541000 -1.534592000

C -1.103345000 2.462549000 -1.876542000

H -1.467068000 3.277857000 -2.495788000

C 0.978578000 2.523412000 -3.317368000

H 1.199525000 1.730345000 -4.043292000

H 1.940507000 2.889590000 -2.936281000

H 0.452163000 3.340041000 -3.820915000

C -1.940750000 2.005815000 -0.839652000

C -3.298574000 2.657287000 -0.628980000

H -3.358812000 3.046019000 0.395480000

H -4.084395000 1.898045000 -0.733046000

H -3.483296000 3.469991000 -1.338193000

C 0.172301000 1.940136000 -2.167469000

O 1.687605000 -1.016700000 0.051776000

O -0.774890000 -1.017476000 1.500155000

C 1.104313000 -2.461772000 1.876935000

H 1.468695000 -3.276917000 2.496049000

C -1.018702000 -2.593814000 3.251243000

H -1.273833000 -1.819174000 3.985900000

H -1.962949000 -2.969374000 2.836752000

H -0.491338000 -3.410502000 3.753664000

C 1.961114000 -1.971381000 0.871761000

C 3.340342000 -2.586978000 0.694233000

H 3.441086000 -2.960425000 -0.332741000

H 4.103622000 -1.810219000 0.831599000

H 3.523952000 -3.404142000 1.398613000

C -0.191879000 -1.974079000 2.135492000

# [Cu^II^(acac)_2_] S = ½ B3LYP*/TZP

Cu -0.002530000 -0.003944000 -0.003914000

O -1.634795000 1.047509000 -0.000513000

O 0.740177000 0.953078000 -1.520565000

C -1.091128000 2.438146000 -1.855732000

H -1.453575000 3.249690000 -2.472281000

C 0.967334000 2.472232000 -3.300703000

H 1.175080000 1.675130000 -4.019019000

H 1.931208000 2.828217000 -2.928443000

H 0.447738000 3.287343000 -3.803492000

C -1.914258000 1.991256000 -0.813544000

C -3.255639000 2.644516000 -0.590370000

H -3.300292000 3.031525000 0.430542000

H -4.040705000 1.889504000 -0.681517000

H -3.446155000 3.452963000 -1.295436000

C 0.170918000 1.905989000 -2.151172000

O 1.666743000 -0.994179000 0.051040000

O -0.773897000 -1.008449000 1.467313000

C 1.093524000 -2.433245000 1.859509000

H 1.457693000 -3.241903000 2.478934000

C -1.029500000 -2.579654000 3.198466000

H -1.296685000 -1.811076000 3.928177000

H -1.962704000 -2.955845000 2.772135000

H -0.506050000 -3.391661000 3.702293000

C 1.947221000 -1.935553000 0.866681000

C 3.325403000 -2.527722000 0.704243000

H 3.439935000 -2.895275000 -0.318477000

H 4.071197000 -1.741683000 0.847671000

H 3.514346000 -3.338942000 1.406665000

C -0.200850000 -1.955752000 2.102770000

# Data Figure 2 and 3.

| ***CSD Refcode*** | ***ANG1 (A)*** | ***ANG2 (A)*** | ***ANG3 (A)*** | ***ANG4 (A)*** | ***DIST1 (D)*** | ***DIST10 (D)*** | ***DIST11 (D)*** | ***DIST12 (D)*** | ***DIST13 (D)*** | ***DIST14 (D)*** | ***DIST15 (D)*** | ***DIST5 (D)*** | ***DIST6 (D)*** | ***DIST7 (D)*** | ***DIST8 (D)*** | ***DIST9 (D)*** |
| --- | --- | --- | --- | --- | --- | --- | --- | --- | --- | --- | --- | --- | --- | --- | --- | --- |
| ACACCU06 | 93.754 | 93.754 | 86.246 | 86.246 | 1.927 | 1.923 | 1.275 | 1.401 | 1.403 | 1.276 | 1.927 | 1.276 | 1.403 | 1.401 | 1.275 | 1.923 |
| ACACCU07 | 92.733 | 92.733 | 87.267 | 87.267 | 1.930 | 1.905 | 1.317 | 1.377 | 1.392 | 1.239 | 1.930 | 1.239 | 1.392 | 1.377 | 1.317 | 1.905 |
| ACACCU08 | 94.006 | 94.006 | 85.994 | 85.994 | 1.904 | 1.908 | 1.285 | 1.401 | 1.382 | 1.297 | 1.904 | 1.297 | 1.382 | 1.401 | 1.285 | 1.908 |
| ACACCU09 | 93.854 | 93.854 | 86.146 | 86.146 | 1.911 | 1.903 | 1.294 | 1.389 | 1.399 | 1.282 | 1.911 | 1.282 | 1.399 | 1.389 | 1.294 | 1.903 |
| ACACCU10 | 93.706 | 93.706 | 86.294 | 86.294 | 1.917 | 1.905 | 1.280 | 1.408 | 1.396 | 1.281 | 1.917 | 1.281 | 1.396 | 1.408 | 1.280 | 1.905 |
| ACACCU11 | 93.625 | 93.625 | 86.375 | 86.375 | 1.914 | 1.900 | 1.278 | 1.412 | 1.395 | 1.280 | 1.914 | 1.280 | 1.395 | 1.412 | 1.278 | 1.906 |
| ACACCU12 | 93.789 | 93.789 | 86.211 | 86.211 | 1.918 | 1.904 | 1.284 | 1.404 | 1.401 | 1.277 | 1.918 | 1.277 | 1.401 | 1.404 | 1.284 | 1.904 |
| ACACCU13 | 93.648 | 93.648 | 86.352 | 86.352 | 1.918 | 1.909 | 1.278 | 1.409 | 1.394 | 1.274 | 1.918 | 1.274 | 1.394 | 1.409 | 1.278 | 1.909 |
| ACACCU14 | 93.617 | 93.617 | 86.383 | 86.383 | 1.919 | 1.908 | 1.276 | 1.415 | 1.395 | 1.275 | 1.919 | 1.275 | 1.395 | 1.415 | 1.276 | 1.908 |
| ACACCU15 | 93.831 | 93.831 | 86.169 | 86.169 | 1.917 | 1.914 | 1.270 | 1.427 | 1.386 | 1.284 | 1.917 | 1.284 | 1.386 | 1.427 | 1.270 | 1.914 |
| ACACCU16 | 93.555 | 93.555 | 86.445 | 86.445 | 1.920 | 1.910 | 1.274 | 1.413 | 1.399 | 1.270 | 1.920 | 1.270 | 1.399 | 1.413 | 1.274 | 1.91 |
| ACACCU17 | 93.694 | 93.694 | 86.306 | 86.306 | 1.914 | 1.911 | 1.276 | 1.417 | 1.392 | 1.278 | 1.914 | 1.278 | 1.392 | 1.417 | 1.276 | 1.911 |
| ACACCU18 | 93.786 | 93.786 | 86.214 | 86.214 | 1.919 | 1.916 | 1.269 | 1.421 | 1.390 | 1.277 | 1.919 | 1.277 | 1.390 | 1.421 | 1.269 | 1.916 |
| ACACCU19 | 93.501 | 93.501 | 86.499 | 86.499 | 1.913 | 1.912 | 1.275 | 1.401 | 1.406 | 1.262 | 1.913 | 1.262 | 1.406 | 1.401 | 1.275 | 1.912 |
| ACACCU20 | 93.636 | 93.636 | 86.364 | 86.364 | 1.915 | 1.898 | 1.285 | 1.409 | 1.399 | 1.265 | 1.915 | 1.265 | 1.399 | 1.409 | 1.285 | 1.898 |
| ACACCU21 | 93.239 | 93.239 | 86.761 | 86.761 | 1.907 | 1.917 | 1.265 | 1.409 | 1.385 | 1.296 | 1.907 | 1.296 | 1.385 | 1.409 | 1.265 | 1.917 |
| ACACCU22 | 93.059 | 93.059 | 86.941 | 86.941 | 1.917 | 1.942 | 1.255 | 1.403 | 1.326 | 1.328 | 1.917 | 1.328 | 1.326 | 1.403 | 1.255 | 1.942 |
| ACACCU23 | 93.418 | 93.418 | 86.582 | 86.582 | 1.929 | 1.927 | 1.267 | 1.439 | 1.386 | 1.280 | 1.929 | 1.28 | 1.386 | 1.439 | 1.267 | 1.927 |
| ACACCU24 | 94.004 | 94.004 | 85.996 | 85.996 | 1.934 | 1.922 | 1.290 | 1.412 | 1.389 | 1.269 | 1.934 | 1.269 | 1.389 | 1.412 | 1.290 | 1.922 |
| ACACCU25 | 94.009 | 94.009 | 85.991 | 85.991 | 1.936 | 1.917 | 1.296 | 1.402 | 1.402 | 1.279 | 1.936 | 1.279 | 1.402 | 1.402 | 1.296 | 1.917 |
| ACACCU26 | 94.007 | 94.007 | 85.993 | 85.993 | 1.936 | 1.918 | 1.292 | 1.395 | 1.411 | 1.280 | 1.936 | 1.280 | 1.411 | 1.395 | 1.292 | 1.918 |
| ACACCU27 | 93.877 | 93.877 | 86.123 | 86.123 | 1.931 | 1.919 | 1.290 | 1.390 | 1.416 | 1.275 | 1.931 | 1.275 | 1.416 | 1.390 | 1.290 | 1.919 |
| ACACCU28 | 94.051 | 94.051 | 85.949 | 85.949 | 1.932 | 1.916 | 1.288 | 1.401 | 1.413 | 1.280 | 1.932 | 1.280 | 1.413 | 1.401 | 1.288 | 1.916 |
| ACACCU29 | 94.024 | 94.024 | 85.976 | 85.976 | 1.930 | 1.916 | 1.293 | 1.397 | 1.414 | 1.275 | 1.930 | 1.275 | 1.414 | 1.397 | 1.293 | 1.916 |
| ACACCU30 | 94.076 | 94.076 | 85.924 | 85.924 | 1.933 | 1.919 | 1.297 | 1.389 | 1.411 | 1.276 | 1.933 | 1.276 | 1.411 | 1.389 | 1.297 | 1.919 |
| ACACCU31 | 94.119 | 94.119 | 85.881 | 85.881 | 1.934 | 1.922 | 1.287 | 1.408 | 1.405 | 1.274 | 1.934 | 1.274 | 1.405 | 1.408 | 1.287 | 1.922 |
| ACACCU32 | 94.093 | 94.093 | 85.907 | 85.907 | 1.932 | 1.921 | 1.290 | 1.400 | 1.410 | 1.274 | 1.932 | 1.274 | 1.410 | 1.400 | 1.290 | 1.921 |
| ACACCU33 | 94.14 | 94.14 | 85.86 | 85.86 | 1.933 | 1.919 | 1.293 | 1.406 | 1.405 | 1.276 | 1.933 | 1.276 | 1.405 | 1.406 | 1.293 | 1.919 |
| ACACCU34 | 94.103 | 94.103 | 85.897 | 85.897 | 1.934 | 1.919 | 1.293 | 1.398 | 1.414 | 1.276 | 1.934 | 1.276 | 1.414 | 1.398 | 1.293 | 1.919 |
| ACACCU35 | 94.102 | 94.102 | 85.898 | 85.898 | 1.930 | 1.920 | 1.293 | 1.397 | 1.418 | 1.275 | 1.930 | 1.275 | 1.418 | 1.397 | 1.293 | 1.920 |
| ACACCU36 | 94.037 | 94.037 | 85.963 | 85.963 | 1.927 | 1.920 | 1.294 | 1.392 | 1.418 | 1.281 | 1.927 | 1.281 | 1.418 | 1.392 | 1.294 | 1.920 |
| ACACCU37 | 94.072 | 94.072 | 85.928 | 85.928 | 1.927 | 1.920 | 1.293 | 1.393 | 1.424 | 1.280 | 1.927 | 1.28 | 1.424 | 1.393 | 1.293 | 1.920 |
| ACACCU38 | 94.084 | 94.084 | 85.916 | 85.916 | 1.929 | 1.919 | 1.294 | 1.388 | 1.430 | 1.282 | 1.929 | 1.282 | 1.430 | 1.388 | 1.294 | 1.919 |
| ACACCU39 | 93.81 | 93.81 | 86.19 | 86.19 | 1.940 | 1.914 | 1.295 | 1.387 | 1.426 | 1.271 | 1.940 | 1.271 | 1.426 | 1.387 | 1.295 | 1.914 |
| ACACCU40 | 93.542 | 93.542 | 86.458 | 86.458 | 1.932 | 1.908 | 1.292 | 1.400 | 1.415 | 1.274 | 1.932 | 1.274 | 1.415 | 1.400 | 1.292 | 1.908 |
| ACACCU | 94.887 | 94.887 | 85.113 | 85.113 | 1.929 | 1.902 | 1.298 | 1.400 | 1.422 | 1.289 | 1.929 | 1.289 | 1.422 | 1.400 | 1.298 | 1.902 |
| ACACCU01 | 93.239 | 93.239 | 86.761 | 86.761 | 1.912 | 1.914 | 1.265 | 1.371 | 1.401 | 1.281 | 1.912 | 1.281 | 1.401 | 1.371 | 1.265 | 1.914 |
| ACACCU02 | 93.606 | 93.606 | 86.394 | 86.394 | 1.917 | 1.919 | 1.207 | 1.395 | 1.395 | 1.272 | 1.917 | 1.272 | 1.395 | 1.395 | 1.270 | 1.919 |
| ACACCU03 | 93.726 | 93.726 | 86.274 | 86.274 | 1.92 | 1.919 | 1.274 | 1.397 | 1.400 | 1.276 | 1.920 | 1.276 | 1.400 | 1.397 | 1.274 | 1.919 |
| ACACCU04 | 93.571 | 93.571 | 86.429 | 86.429 | 1.923 | 1.921 | 1.268 | 1.404 | 1.407 | 1.267 | 1.923 | 1.267 | 1.407 | 1.404 | 1.268 | 1.921 |
| ACACCU05 | 93.617 | 93.617 | 86.383 | 86.383 | 1.926 | 1.922 | 1.283 | 1.397 | 1.402 | 1.279 | 1.926 | 1.279 | 1.402 | 1.397 | 1.283 | 1.922 |
| COQDUH | 93.529 | 93.228 | 86.699 | 86.627 | 1.911 | 1.904 | 1.273 | 1.344 | 1.373 | 1.278 | 1.899 | 1.271 | 1.386 | 1.385 | 1.273 | 1.917 |
| COQDUH01 | 92.027 | 92.458 | 178.925 | 175.643 | 1.916 | 1.907 | 1.292 | 1.389 | 1.395 | 1.294 | 1.908 | 1.292 | 1.367 | 1.389 | 1.280 | 1.906 |
| HAMGEH | 93.211 | 93.211 | 86.789 | 86.789 | 1.922 | 1.923 | 1.263 | 1.393 | 1.387 | 1.276 | 1.922 | 1.276 | 1.387 | 1.393 | 1.263 | 1.923 |
| JIRKUR | 93.482 | 93.482 | 86.518 | 86.518 | 1.926 | 1.929 | 1.262 | 1.384 | 1.396 | 1.249 | 1.926 | 1.249 | 1.396 | 1.384 | 1.262 | 1.929 |
| JIRKUR | 93.714 | 93.714 | 86.286 | 86.286 | 1.911 | 1.929 | 1.266 | 1.400 | 1.395 | 1.253 | 1.911 | 1.253 | 1.395 | 1.400 | 1.266 | 1.929 |
| VOLTIA | 93.591 | 93.591 | 86.409 | 86.409 | 1.912 | 1.912 | 1.262 | 1.375 | 1.394 | 1.250 | 1.912 | 1.250 | 1.394 | 1.375 | 1.262 | 1.912 |
| ZUFRID | 93.65 | 93.65 | 86.35 | 86.35 | 1.919 | 1.917 | 1.278 | 1.386 | 1.390 | 1.272 | 1.919 | 1.272 | 1.390 | 1.386 | 1.278 | 1.917 |
| SESTAO | 93.91 | 93.91 | 180 | 179.98 | 1.915 | 1.915 | 1.278 | 1.388 | 1.388 | 1.278 | 1.915 | 1.278 | 1.388 | 1.388 | 1.278 | 1.915 |
